# Supplementary material for: Developing a core outcome set for interventions in people with mild cognitive impairment: study protocol
Source: BMJ Open. 2025 Jan 20;15(1):e090818. doi: 10.1136/bmjopen-2024-090818 (PMC11751846; doi:10.1136/bmjopen-2024-090818)
Supplement: online supplemental file 1 [file bmjopen-15-1-s001.docx]

| **Core Outcome Set-STAndardised Protocol (COS-STAP) Items** | | | **Location** |
| --- | --- | --- | --- |
| ABSTRACT | | | |
| Title | 1a | Identify in the title that the paper describes the protocol for the planned development of a COS | Title |
| Abstract | 1b | Provide a structured abstract | Abstract |
| INTRODUCTION | | | |
| Background and objectives | 2a | Describe the background and explain the rationale for developing the COS, and identify the reasons why a COS is needed and the potential barriers to its implementation | Introduction: Background and objectives |
|  | 2b | Describe the specific objectives with reference to developing a COS | Introduction: Background and objectives |
| Scope | 3a | Describe the health condition(s) and population(s) that will be covered by the COS | Introduction: Scope |
|  | 3b | Describe the intervention(s) that will be covered by the COS | Introduction: Scope |
|  | 3c | Describe the context of use for which the COS is to be applied | Introduction: Scope |
| METHODS | | | |
| Stakeholders | 4 | Describe the stakeholder groups to be involved in the COS development process, the nature of and rationale for their involvement and also how the individuals will be identified; this should cover involvement both as members of the research team and as participants in the study | Methods: Stakeholders |
| Information sources | 5a | Describe the information sources that will be used to identify the list of outcomes. Outline the methods or reference other protocols/papers | Methods: Information sources |
|  | 5b | Describe how outcomes may be dropped/combined, with reasons | Methods: Information sources (Stage 3) |
| Consensus process | 6 | Describe the plans for how the consensus process will be undertaken | Methods: Information sources (Stage 5) |
| Consensus definition | 7a | Describe the consensus definition | Methods: Information sources (Stage 5) |
|  | 7b | Describe the procedure for determining how outcomes will be added/combined/dropped from consideration during the consensus process | Methods: Information sources (Stage 5) |
| ANALYSIS | | | |
| Outcome scoring/feedback | 8 | Describe how outcomes will be scored and summarised, describe how participants will receive feedback during the consensus process | Methods: Information sources (Stage 4) |
| Missing data | 9 | Describe how missing data will be handled during the consensus process | Methods: Information sources (Stage 4) |
| ETHICS and DISSEMINATION | | | |
| Ethics approval/informed consent | 10 | Describe any plans for obtaining research ethics committee/institutional review board approval in relation to the consensus process and describe how informed consent will be obtained (if relevant) | Ethics and dissemination: Ethics approval/informed consent |
| Dissemination | 11 | Describe any plans to communicate the results to study participants and COS users, inclusive of methods and timing of dissemination | Ethics and dissemination: Dissemination |
| ADMINISTRATIVE INFORMATION | | |  |
| Funders | 12 | Describe sources of funding, role of funders | Funding |
| Conflicts of interest | 13 | Describe any potential conflicts of interest within the study team and how they will be managed | Conflicts of interest |
