# Supplementary material for: Developing a core outcome set for interventions in people with mild cognitive impairment: study protocol
Source: BMJ Open. 2025 Jan 20;15(1):e090818. doi: 10.1136/bmjopen-2024-090818 (PMC11751846; doi:10.1136/bmjopen-2024-090818)
Supplement: Supplementary file 2 [file bmjopen-2024-090818.reviewer_comments.pdf]

PEER REVIEW HISTORY

BMJ Open publishes all reviews undertaken for accepted manuscripts. Reviewers are asked to complete a checklist review form and are provided with free text boxes to elaborate on their assessment. These free text comments are reproduced below.

ARTICLE DETAILS

Title (Provisional)

Developing a core outcome set for interventions in people with mild cognitive impairment: study protocol

Authors

Gabb, Victoria Grace; Harding, Sam; McNair, Angus GK; Clayton, Julie; Barrett-Muir, Winsome; Richardson, Alan; Woodward, Natalie; Alderman , Sophie; Dooley, Jemima; Webb, Joseph; Rudd, Sarah; coulthard, elizabeth; Turner, Nicholas

---

VERSION 1 - REVIEW

Reviewer 1

Name Osaki, Tohmi

Affiliation Kobe Gakuin University Faculty of Rehabilitation, Department of Occupational Therapy

Date 12-Sep-2024

COI None

I am honoured to have the opportunity to review this excellent manuscript. The protocol is appropriately designed and well-written by the guidelines. I am confident that it can be published without major revisions and will significantly contribute to our understanding of the outcomes for MCI.

I want to give my opinion on the appropriate outcomes of research on people with MCI, which this study will clarify. Reversion from MCI to cognitively normal is possible, but unfortunately, a certain percentage of patients convert to dementia. Therefore, even in the MCI phase, it is necessary to evaluate the patient with a view to future dementia care and delaying the progression of dementia. What is essential in this process is the awareness of the family or care staff regarding the care of the person with MCI and how this awareness may change. There may not be a sufficient number of research reports on so-called 'positive aspects of care', but it is necessary to pay attention to this. Also, in addition to cognitive function and biomarkers, lifestyle change, i.e. whether or not risk reduction has been achieved, is an essential outcome for people with MCI. Lifestyle modification may be effective not only in reducing the conversion rate to dementia in the future but also in

delaying the progression of dementia. Furthermore, as some people with MCI are in middle age, performance and engagement in work activities are also important. Outcomes need to be considered according to age and the person's social role.

Through this research, your research team will also clarify the abovementioned issues. I look forward to reading the results of the study.

**Reviewer**	**2**
**Name**	**Wang, Lina**
**Affiliation**	**Huzhou University, School of Nursing**
**Date**	**11-Oct-2024**
**COI**	**None**

This study will be the first to develop a COS for evaluating interventions in populations with MCI. At present, research and clinical practice related to MCI lack standardized outcome reporting, which hampers comparison and synthesis across studies. The development and implementation of a COS will enable researchers and clinicians to report patient-centered outcomes with greater transparency and consistency, enhancing both the quality of research and the utility of clinical decision-making. The study carries significant clinical implications; however, certain sections of the manuscript require further refinement:

1. The background section underscores the importance and urgency of creating a COS for MCI interventions. MCI outcomes are inherently tied to specific symptom clusters, which in turn are closely linked to the choice of intervention strategies. However, the manuscript does not discuss these symptom clusters or the existing interventions for MCI patients. This omission makes it challenging to identify the preliminary structure or theoretical framework underlying the COS, raising concerns about the scientific validity of its conceptual development. Further elaboration in this area is necessary.

2. As noted by the authors, the COS will be applicable to both pharmacological and non-pharmacological interventions, implying that it may need to incorporate subsets tailored to different intervention strategies or MCI pathological subtypes. To provide meaningful, context-specific guidance, the COS framework should account for such differences. Consequently, exploratory analyses of potential subgroups should be designed and conducted at various stages of the study—systematic reviews, interviews, and evidence synthesis—to assess their impact on the construction of the COS.

3. In the methodology section, the description of methodological design across the various study stages is overly simplistic. For instance, during the umbrella systematic review phase, the development of the search strategy and the inclusion of comprehensive databases, such as PubMed and Web of Science, require further detail in the protocol. Although the authors mention involving experts and patients (PPI) in co-developing interview guides, the “themes

and scope of the interviews" remain underdeveloped. Providing a more thorough explanation of these aspects in the research design would help readers better evaluate the study's methodological rigor and accuracy.

VERSION 1 - AUTHOR RESPONSE

Reviewer: 1
Dr. Tohmi Osaki, Kobe Gakuin University Faculty of Rehabilitation
Comments to the Author:
I am honoured to have the opportunity to review this excellent manuscript. The protocol is appropriately designed and well-written by the guidelines. I am confident that it can be published without major revisions and will significantly contribute to our understanding of the outcomes for MCI.
I want to give my opinion on the appropriate outcomes of research on people with MCI, which this study will clarify. Reversion from MCI to cognitively normal is possible, but unfortunately, a certain percentage of patients convert to dementia. Therefore, even in the MCI phase, it is necessary to evaluate the patient with a view to future dementia care and delaying the progression of dementia. What is essential in this process is the awareness of the family or care staff regarding the care of the person with MCI and how this awareness may change. There may not be a sufficient number of research reports on so-called 'positive aspects of care', but it is necessary to pay attention to this. Also, in addition to cognitive function and biomarkers, lifestyle change, i.e. whether or not risk reduction has been achieved, is an essential outcome for people with MCI. Lifestyle modification may be effective not only in reducing the conversion rate to dementia in the future but also in delaying the progression of dementia. Furthermore, as some people with MCI are in middle age, performance and engagement in work activities are also important. Outcomes need to be considered according to age and the person's social role.
Through this research, your research team will also clarify the abovementioned issues. I look forward to reading the results of the study.

We would like to sincerely thank Dr Osaki for their support of the study and positive feedback regarding our manuscript. We agree that it is important to consider that MCI has an uncertain trajectory, and often uncertain aetiology, and that this uncertainty and risk of progression needs to be fully considered. We aim to recruit participants who do and do not have biomarkers of Alzheimer's disease and other neurodegenerative conditions to ensure their experiences are reflected in the COS. We anticipate that the outcomes highlighted by Dr Osaki will be considered for inclusion in the COS.

Reviewer: 2
Dr. Lina Wang, Huzhou University
Comments to the Author:
This study will be the first to develop a COS for evaluating interventions in populations with MCI. At present, research and clinical practice related to MCI lack standardized outcome reporting, which hampers comparison and synthesis across studies. The development and implementation of a COS will enable researchers and clinicians to report patient-centered outcomes with greater transparency and consistency, enhancing both the quality of research and the utility of clinical decision-making. The study carries significant clinical implications; however, certain sections of the manuscript require further refinement:

We would like to thank Dr Wang for their insightful feedback.

1. The background section underscores the importance and urgency of creating a COS for MCI interventions. MCI outcomes are inherently tied to specific symptom clusters, which in turn are closely linked to the choice of intervention strategies. However, the manuscript does not discuss these symptom clusters or the existing interventions for MCI patients. This omission makes it challenging to identify the preliminary structure or theoretical framework underlying the COS, raising concerns about the scientific validity of its conceptual development. Further elaboration in this area is necessary.

Thank you for this important feedback. We have made several changes after reflection on your comments, which we believe have improved the manuscript.

a) ***We have changed the introductory paragraph to elaborate on the different symptom clusters of mild cognitive impairment (i.e., amnestic vs. non-amnestic, single domain vs. multi-domain).***
b) ***In the Methods section, we have clarified our intention to create a COS that is relevant across subtypes and intervention types, and that we anticipate there will be outcomes specific to intervention types and/or subtypes of MCI.***
c) ***Finally in the Discussion section, we have added a reflection on our decision to enhance the applicability of the COS to all intervention types and subtypes of MCI, recognising that this means more granular outcomes relating to specific interventions or subtypes of MCI may not be included.***

We understand the author's concerns regarding a theoretical framework. However, rather than a 'top-down' deductive approach of using a theoretical framework to develop the COS, the study is designed as a 'bottom-up' inductive approach, starting with observations/data from the review and interviews which will then be iteratively developed into a COS through the surveys and consensus meeting(s). This approach means we are learning from those with lived and/or professional

experience and they guide the COS development throughout the study. Once outcomes have been identified, we intend to add structure through applying a framework and anticipate this to be Dodd's taxonomy with additional subcategories (e.g., cognitive functioning may be split up more than in Dodd's taxonomy), if this is seen as acceptable by stakeholders and after discussion with the PPIE group.

2. As noted by the authors, the COS will be applicable to both pharmacological and non-pharmacological interventions, implying that it may need to incorporate subsets tailored to different intervention strategies or MCI pathological subtypes. To provide meaningful, context-specific guidance, the COS framework should account for such differences. Consequently, exploratory analyses of potential subgroups should be designed and conducted at various stages of the study—systematic reviews, interviews, and evidence synthesis—to assess their impact on the construction of the COS.
When developing a COS, decisions need to be made regarding whom the COS will be relevant for (population) and in what situations (intervention). The idea of the core outcome set is to develop a minimum set of outcomes agreed important for MCI, but we recognise that this may mean more specific outcomes relating to a given intervention type or underlying pathology may not be included- though researchers are encouraged to supplement the COS with any additionally relevant or specific outcomes for their own study. The COS is intended to be a starting point. Accordingly, we have added a limitation to the discussion section in light of the risk that highly specific outcomes may not be included.

We decided to generate a COS which is applicable to MCI without specifying subtypes because often patients and/or researchers do not have access to, or funding for, expensive scans and testing which could identify underlying pathology, and often tests do not identify a known cause. We believe this approach makes the study more inclusive and will mean the COS has wider impact. . We will include individuals with different and unknown aetiologies to ensure those most relevant to MCI as a whole are included. A similar approach was recently adopted for the multiple sclerosis core outcome set, included pharmacological and non-pharmacological interventions, and included patients with different subtypes of multiple sclerosis. .

For the review, we have intentionally decided to include outcomes from across intervention types, as we anticipate there will be some specific to given intervention types – though this does not mean they won't be relevant in other interventions. We will report study characteristics and compare whether there were specific outcomes which were only reported in studies of a particular intervention.

3. In the methodology section, the description of methodological design across the various study stages is overly simplistic. For instance, during the umbrella systematic review phase, the development of the search strategy and the inclusion of comprehensive databases, such as PubMed and Web of Science, require further detail in the protocol. Although the authors mention involving experts and patients (PPI) in co-developing interview guides, the "themes and scope of the interviews" remain underdeveloped. Providing a more thorough explanation of these aspects in the research design would help readers better evaluate the study's methodological rigor and accuracy.

Thank you for this feedback on adding detail to our methodology section. We have made several improvements to the manuscript in light of your comments:

a) **Further information on the content of the interviews and what will be explored in the thematic analysis has been added.**
b) **Further detail has been added to the review section of the methodology on inclusion of different subtypes and interventions and details of the search strategy.**
c) **Detail has been added to the search strategy**
d) **Additional information on the influence of PPI group and PPI co-applicants has been added.**
